# Partial weight‐bearing and range of motion limitation significantly reduce the loads at medial meniscus posterior root repair sutures in a cadaveric biomechanical model

**DOI:** 10.1002/ksa.12465

**Published:** 2024-09-17

**Authors:** Matthias Sukopp, Nina Schwab, Jonas Schwer, Julian Frey, Jonas Walter Metzger, Anita Ignatius, Mario Perl, Firooz Salami, Daniel Vogele, Thomas Kappe, Andreas Martin Seitz

**Affiliations:** ^1^ Institute of Orthopaedic Research and Biomechanics University Hospital Ulm Ulm Germany; ^2^ Department of Trauma and Orthopedic Surgery, University Hospital Erlangen Friedrich‐Alexander University Erlangen‐Nürnberg (FAU) Erlangen Germany; ^3^ Movement Analysis Laboratory, Department of Orthopaedics and Trauma Surgery Heidelberg University Hospital Heidelberg Germany; ^4^ Department of Diagnostic and Interventional Radiology University Hospital Ulm Ulm Germany; ^5^ Department of Orthopaedic Surgery RKU University Hospital Ulm Ulm Germany

**Keywords:** dynamic knee joint simulator, knee contact mechanics, medial meniscus posterior root avulsion, rehabilitation protocol, suture repair

## Abstract

**Purpose:**

The aim of this study was to investigate the influence of medial meniscus posterior root avulsion (MMPRA) before and after surgical treatment on the biomechanics of the knee joint, including suture repair forces during daily and crutch‐assisted gait movements.

**Methods:**

MMPRA were investigated in eight human cadaver knee joint specimens by a dynamic knee joint simulator with daily (normal gait, gait with additional rotational movement, standing up, sitting down) and rehabilitation‐associated movements (crutch‐assisted gait with limited flexion range of motion [30°] and 30% [toe‐touch weight‐bearing, TTWB] and 50% of body weight [partial weight‐bearing, PWB]) with simulated physiologic muscle forces. Each specimen was tested in intact, torn and repaired (transtibial suture) state. The biomechanical parameters were: medial mean contact pressure and area, knee joint kinematics, medial displacement of the posterior meniscus horn and loading on the anchoring suture.

**Results:**

Significant reduction of the contact area due to the avulsion was observed in all movements except for PWB and sitting down. MMPRA repair significantly increased the contact areas during all movements, bringing them to levels statistically indistinguishable from the initial state. MMPRA resulted in a medial displacement up to 12.8 mm (sitting down) and could be reattached with a residual displacement ranging from 0.7 mm (PWB) to 5.7 mm (standing up), all significantly (*p* < 0.001) reduced compared to the torn state. The mean peak anchoring suture load increased from TTWB (77 N), PWB (91 N) to normal gait (194 N), gait rotation (207 N), sitting (201 N; *p* < 0.01) and to standing up (232 N; *p* = 0.03).

**Conclusion:**

Surgical treatment of MMPRA allows restoration of physiological knee joint biomechanics. Crutch‐assisted movements reduce the loading of the repair suture, thus likewise the risk for failure. From a biomechanical point of view, crutch‐assisted movements are recommended for the early rehabilitation phase after MMPRA repair.

**Level of Evidence:**

Level V.

**Abbreviations**
BMI: body mass index
BW: body weight
FWB: full weight‐bearing
MMPRA: medial meniscus posterior root avulsion
PWB: partial weight‐bearing
ROM: range of motion
RSA: roentgen stereophotogrammetric analysis
SD: standard deviation
TTWB: toe‐touch weight‐bearing
Yrs: years

## INTRODUCTION

Knee menisci are intra‐articular fibro‐cartilaginous structures, which are essential for knee joint homeostasis by providing multiple functions in knee joint biomechanics and kinematics [40]. Traumatic meniscus injuries are, with an incidence of 60–80 per 100,000 per year, one of the most common acute knee injuries [20, 39, 51]. In particular, complete and unstable radial meniscus tears result in a loss of the ability to convert axial loads into circumferential hoop stress that is necessary for equal pressure distribution on the tibial plateau [67]. Likewise, a complete radial tear located within 9 mm of the posterior medial meniscus root (LaPrade type 2; [32]), which constitutes up to 20% of all meniscal pathologies [16], can lead up to a 2.5‐fold increase in tibiofemoral contact pressure [68], and consequently to an early onset of knee joint osteoarthritis (OA) [1, 33, 45, 53, 60].

A medial meniscus posterior root avulsion (MMPRA) also significantly affects the knee joint kinematics by increasing the knee joint's external tibial rotation ability, causing instability and an associated higher risk for additional knee joint injuries [1, 17, 41, 44]. A current surgical treatment option of MMPRA is the transtibial pull‐out repair [13, 65], which is able to restore the tibiofemoral contact pressure and the knee joint kinematics comparable to the initial native state [24, 25, 31, 35, 37, 68]. Clinically, MMPRA repair leads to reduced meniscus extrusion, a significant improvement in functional knee scores, and an associated slowing of OA progression [19, 37]. Even so, adequate surgical treatment must be combined with a rehabilitation protocol that meets the patient's postoperative status including the reduction of postoperative failure, such as incomplete healing and associated retearing (32.6%) and suture failure (4.97%) [11]. However, there is neither a clinical consensus nor any objective biomechanical data available for the postoperative treatment of MMPRA [19, 26, 28, 49, 52, 55, 56]. In principle, physiotherapists and clinicians make a distinction between an accelerated and a restricted rehabilitation protocol: While an accelerated rehabilitation protocol involves early postoperative weight‐bearing under full weight‐bearing (FWB) application with unrestricted range of motion (ROM), a restrictive rehabilitation protocol applies conservative weight‐bearing that typically ranges from crutch‐assisted toe‐touch weight‐bearing (TTWB = 30% body weight [BW]) to partial weight‐bearing (PWB = 50% BW) and also restricts the postoperative ROM [26]. The aim of adequate rehabilitation is to avoid high shear and compressive loads that could potentially impair the healing process after MMPRA repair. By contrast, extensive movement and loading restrictions are associated with a higher risk for joint stiffness and muscular atrophy [4, 26, 29, 66]. However, the biomechanical impact of restrictive or accelerated rehabilitation is still unknown [49].

Given the significant impact of the early postoperative phase after MMPRA repair, our first goal is to identify the impact of MMPRA on knee joint biomechanics and kinematics before and after surgical repair. Since there is no data available that objectively describes the biomechanical differences when applying either an unrestricted or conservative rehabilitation regime after MMPRA repair, we investigated the impact of different movement simulations on the most important biomechanical measures. On the basis of the current literature, we hypothesized that

1) an MMPRA causes a decreased tibiofemoral contact area and increased mean tibiofemoral contact pressure, which can be restored to the native state by MMPRA repair; and
2) the simulation of crutch‐assisted movements that are associated with a restrictive rehabilitation protocol results in lower MMPRA suture repair forces and associated biomechanical parameters compared to unrestricted movements that represent an accelerated rehabilitation protocol.

## METHODS

### Specimen preparation

Based on a previous study [48] investigating the maximum rupture force at the native meniscus root (360 ± 168 N) compared with the maximum suture force of a locking loop suture (191 ± 45 N). Consequently, an a priori power analysis was performed using G*Power (Wilcoxon signed‐rank test; dz = 1.12; *δ* = 2.9; critical *t* = 1.96; df = 5.7) to identify *n* = 7 as the sample size necessary for the present study with a power of 0.81 at the significance level (=α err prob) of *p* < 0.05. In order to even further improve the power of the study, the total sample size was increased to *n* = 8. On that, the soft tissues of eight fresh‐frozen human cadaver knee joint specimens (four right, four left, six male, two female, age: 51 ± 12 yrs, body mass index [BMI]: 22 ± 4 kg/m^2^; IRB 326/19, Ulm University) were removed while the joint capsule, ligamentous structures and menisci were preserved. The exclusion criteria for cadaveric specimens were: age >65 years, any history of anterior cruciate ligament, meniscus or patella surgery, presence of OA, and existing meniscus pathologies. Subsequently, the femur and tibia diaphyses were physiologically aligned and potted in customized metal pots using polymethylmethacrylate (Technovit 3040®, Kulzer GmbH). To be able to adequately transfer the physiological, highly dynamic muscle forces (Table 1) from the electromechanical actuators to the knee joint, steel cables were directly connected to bicortically anchored bone screws which were inserted at the midpoint of each respective muscle [59]. After initial preparation, the cadavers were stored at −20°C until the testing. After gradual overnight thawing at the day of testing a posteromedial capsulotomy was performed to reproducibly set and repair the MMPRA (LaPrade type 2 [32]). An additional anteromedial capsulotomy allowed for positioning of a pressure‐sensitive film. Tear simulation using a scalpel and consecutive surgical transtibial pull‐out repair [13, 53] using a braided fibre wire (EP 3.5, USP 0, Pearsalls Ltd.) was performed by an experienced senior consultant (TK; Details: Supporting Information S1: 1A). The knee joints were continuously kept moist using saline‐saturated gauze.

**Table 1:** Applied maximum quadriceps muscle force in N, consisting of the simultaneous simulation of the Vastus medialis, Rectus femoris and Vastus lateralis and the according time in s to reach this force.

Movement	Maximum quadriceps force (N)	Time (s)
TTWB	116	0.5
PWB	176	0.5
Gait	728	0.6
GaitRotation	887	0.6
SitToStand	1835	0.5
StandToSit	1402	2.9

### Dynamic knee joint simulator

An established dynamic knee joint simulator [59] was updated by utilizing seven electromagnetic actuators (PS10‐70x320U‐BL‐QJ, LinMot GmbH®) to simulate the major knee‐spanning muscles. Following an established workflow, daily movements like ground‐level walking (Gait), ground‐level walking with an associated 15° ankle rotation (GaitRotation), standing up (SitToStand), sitting down (StandToSit) and crutch‐assisted gait under 30% BW (TTWB) and 50% BW (PWB) with a limited ROM of max. 30° flexion [25] were conducted to assess the individual muscle‐force‐over‐time profiles. Therefore, before this study, a combined motion analysis and inverse dynamic study on 13 healthy volunteers without knee pathologies (age: 26 ± 2 yrs, weight: 65 ± 12 kg, height = 1.8 ± 0.1 m) [59] was conducted. All volunteers gave their written informed consent before the study (IRB S‐832/2019, Heidelberg University). The evaluated muscle‐force‐over‐time profiles served as input for each respective muscle simulation. A time‐triggered control between knee flexion and the corresponding muscle force simulation was implemented for each movement.

### Data acquisition

**Figure 1:**
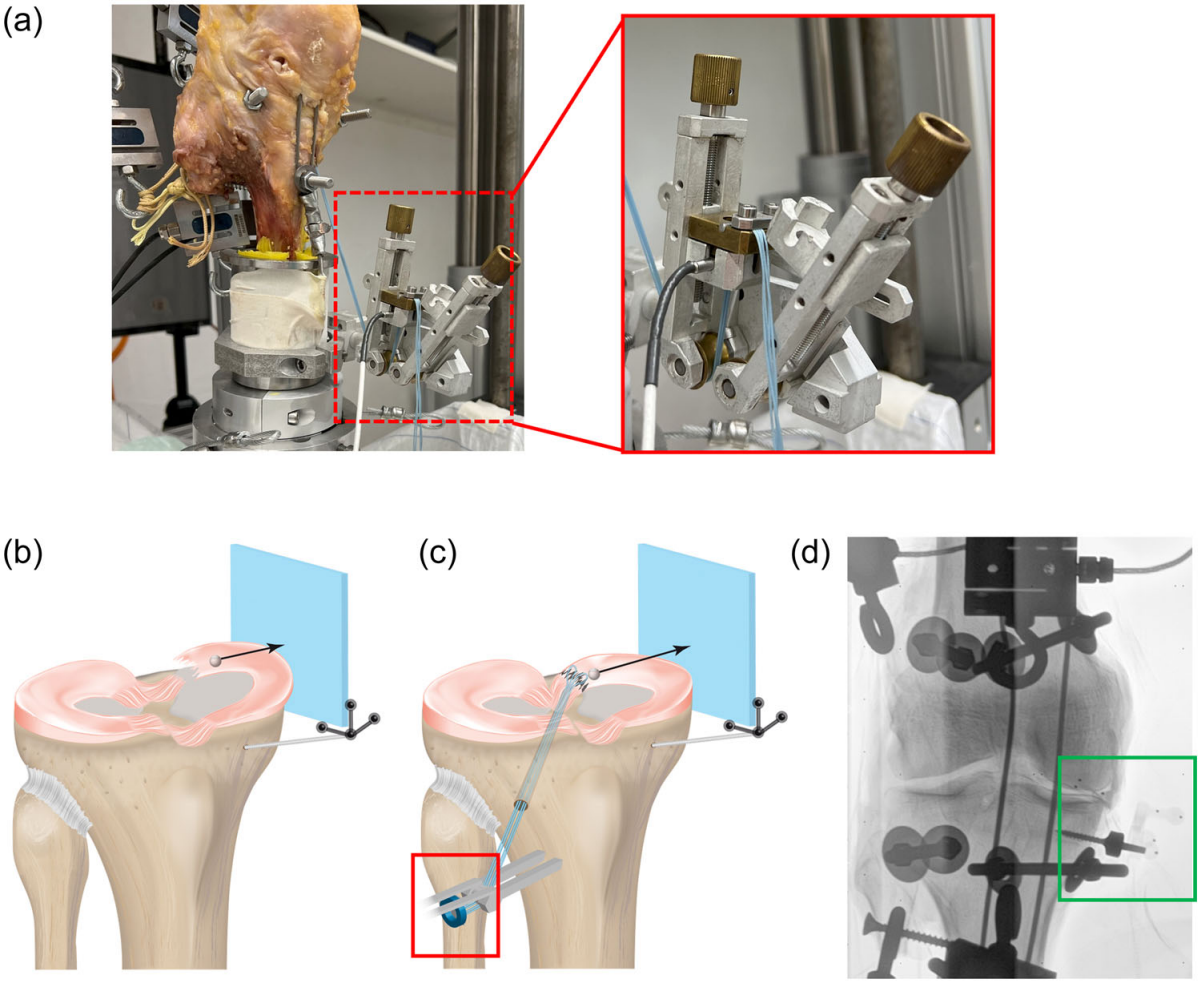
(a) Ring force transducer, fixed in a custom‐made holder to enable adequate suture pre‐tension. Illustration of a torn (b) and repaired (c) posterior horn with medially translated meniscus body and adapted ring force transducer (red rectangle) and (d) radiograph image with coordinate system and tantalum marker beads in the meniscus body (green rectangle).

The tibiofemoral mean contact pressure and area were simultaneously measured between the medial tibial plateau and the medial meniscus using an equilibrated and calibrated pressure‐sensitive film (K‐scan sensor 4000®, sensitivity 10.342 kPa; Tekscan Inc.) [61] and analyzed using corresponding software (I‐scan 6.03, Tekscan Inc.). To prevent the pressure‐sensitive film from moving, it was attached to the tibia with a screw. A motion‐tracking system consisting of six cameras (Prime13, OptiTrack®, NaturalPoint, Inc.) was utilized to capture the knee joint kinematics, while two passive reflective rigid body markers were aligned with the bony landmarks of the femur and tibia diaphyses. Calibration of the tracking system was performed in accordance with the manufacturer's guidelines and motion recording was achieved using developer software (Motive 4.0; OptiTrack). Subsequently, the kinematic parameters of each rigid body were incorporated into a customized analysis protocol (Matlab, The MathWorks) while the movement was analysed according to Grood and Suntay [15]. The ROM of tibial rotation during the movement cycle was partitioned into internal rotation (negative denoted) and external rotation (positive denoted). Maximum forces acting on the suture fixation of the posterior root, thus identifying critical movements which may result in impaired healing [65], were captured using a special force transducer (Figure 1a; Wazau Mess‐Prüfsysteme GmbH). No suture re‐tensioning was performed between the various movements to ensure comparability. However, a visual check was carried out after each movement to ensure sufficient suture fixation. The medial displacement [21] of the meniscus posterior horn was compared to the intact state following MMPRA and subsequent repair by means of roentgen stereophotogrammetric analysis (RSA). A 1 mm diameter tantalum marker bead (RSA Biomedical AB) was injected into the posterior aspect, close to the dissected root through the posterior window using a special RSA bead inserter (Halifax Biomedical Inc.). The methodology employed in this study involved the application of the orthogonal projection technique (Figure 1b–d; Details: Supporting Information S1: 1B).

### Biomechanical testing

**Figure 2:**
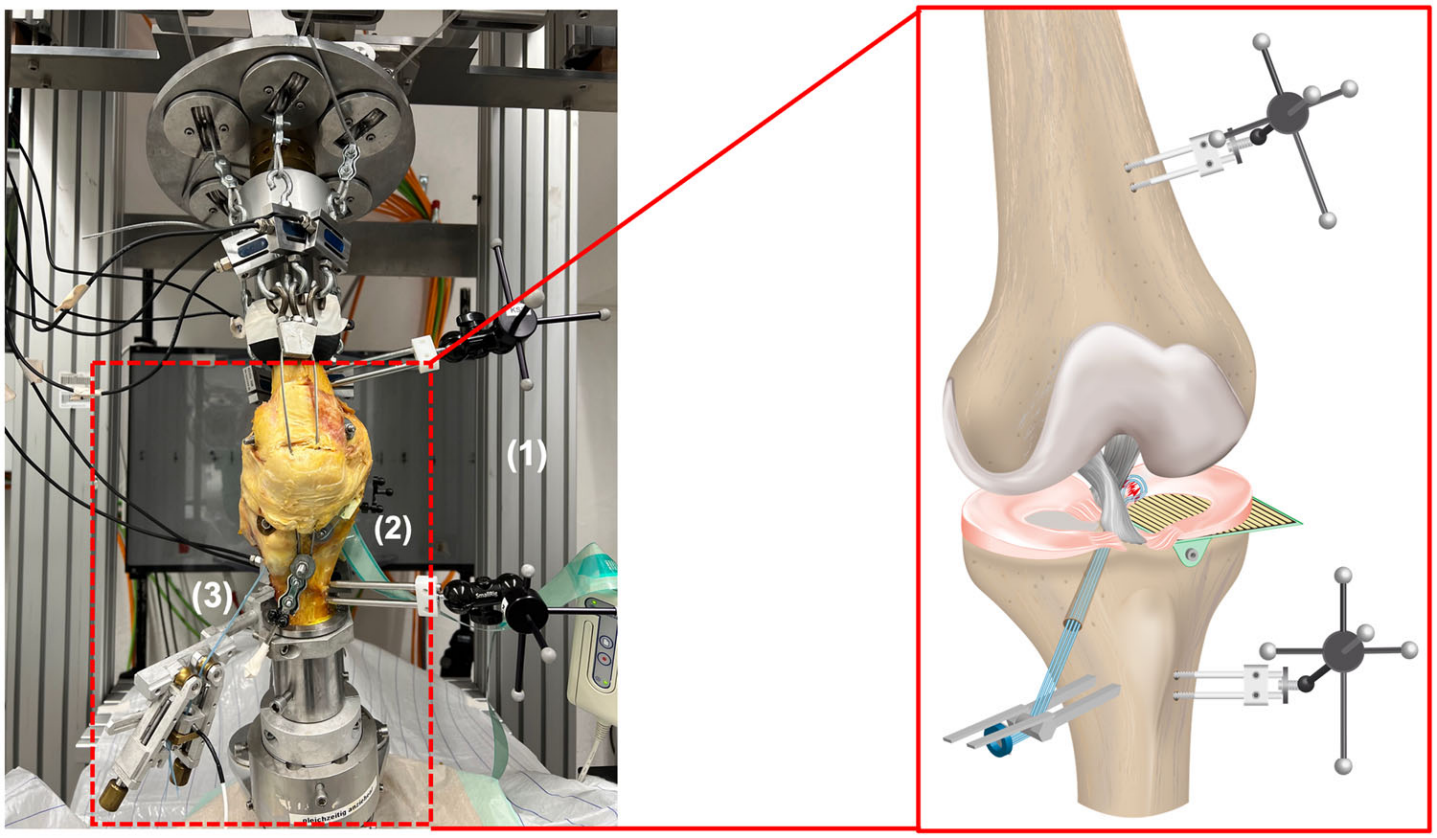
(a) Dynamic knee simulator with embedded specimen, muscle simulation via steel cables, and (1) passive reflective rigid marker bodies (OptiTrack®, NaturalPoint, Inc.) at the femur and tibia, (2) Tekscan® pressure‐sensitive film (K‐scan sensor 4000, Tekscan, Inc.) and (3) ring force transducer (Wazau Mess‐Prüfsysteme GmbH). (b) Schematic drawing of the biomechanical test configuration.

Each specimen underwent testing in the dynamic knee simulator (Figure 2) in three consecutive meniscal states: intact, torn (LaPrade type 2) and repaired with a double double‐locking loop (D‐DLL) transtibial pull‐out repair [34]. For each meniscal state, a total of six movements were simulated in a randomized order: TTWB, PWB, Gait, GaitRotation, standing up (SitToStand) and sitting down (StandToSit). Each exercise was repeated six times, while only the last evaluable repetition was used for results analyses.

### Statistical analysis

The normal distribution of the results data was checked using the Shapiro–Wilk test. Normally distributed data were evaluated by repeated measure one‐way analysis of variance (ANOVA) and Tukey's multiple comparison test and values are given with mean ± SD. Non‐normally distributed data were evaluated by Friedmann testing. Results are presented with mean and standard deviations or median and min/max. Outlier tests were performed on RSA evaluation and the optically recorded kinematic parameters to exclude errors caused by optical distortions. Subsequently, ANOVA with mixed‐effects analysis models were conducted. All statistical tests and the corresponding plots were performed using a statistical software package (GraphPad Prism® 8.4.3, GraphPad Software). *p* ≤ 0.05 was considered to be statistically significant.

## RESULTS

### Medial tibiofemoral mean contact pressure

**Figure 3:**
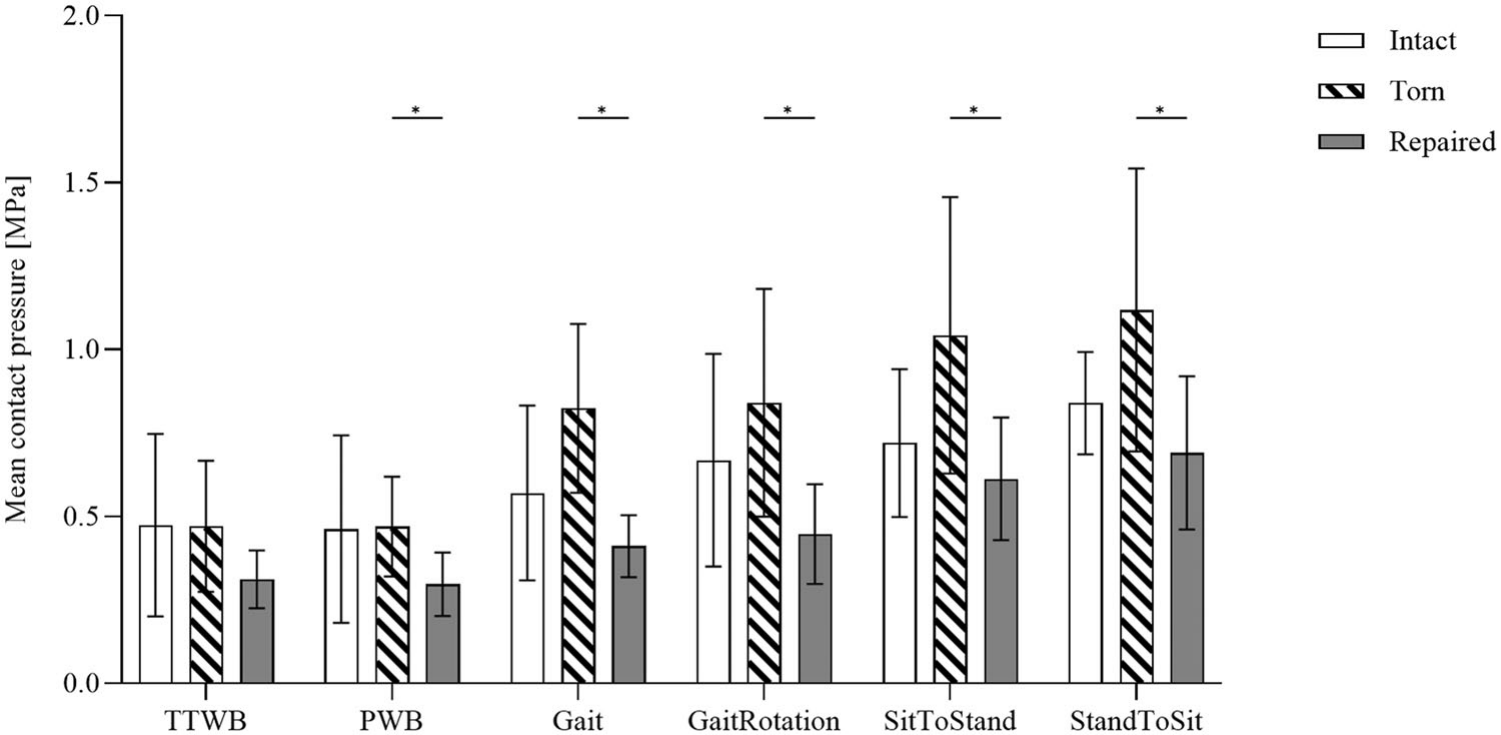
Mean contact pressure (mean ± SD) measurements during six movements (four‐point crutch gait under 30% BW [TTWB] and 50% BW [PWB], Gait, gait with an associated 15° ankle rotation [GaitRotation], standing up [SitToStand] and sitting down [StandToSit]), for the intact (white bar), torn (hatched bar) and repaired (solid grey bar) meniscal state. One‐way analysis of variance and Tukey's multiple comparison test. BW, body weight; PWB, partial weight‐bearing; TTWB, toe‐touch weight‐bearing. *p ≤ 0.05; *n* = 8.

Mean contact pressure measurements in the intact meniscal state demonstrated a progressive increase from the crutch‐assisted PWB gait pattern of up to 82% when compared to the sitting movement (Figure 3; Statistical details: Supporting Information S2: 2A). Subsequent to MMPRA simulation, the two crutch‐assisted gait patterns indicated the lowest mean contact pressure measurements with no differences when compared to the intact state. Under gait simulation, the mean contact pressure increased by 44% to 0.82 MPa, as well as standing up and sitting down simulation by 45% and 33%, respectively. MMPRA repair significantly reduced mean contact pressures in all movements (*p* < 0.03), with the exception of TTWB simulation, resulting in contact pressure measurements similar to the intact state.

### Medial tibiofemoral contact area

**Figure 4:**
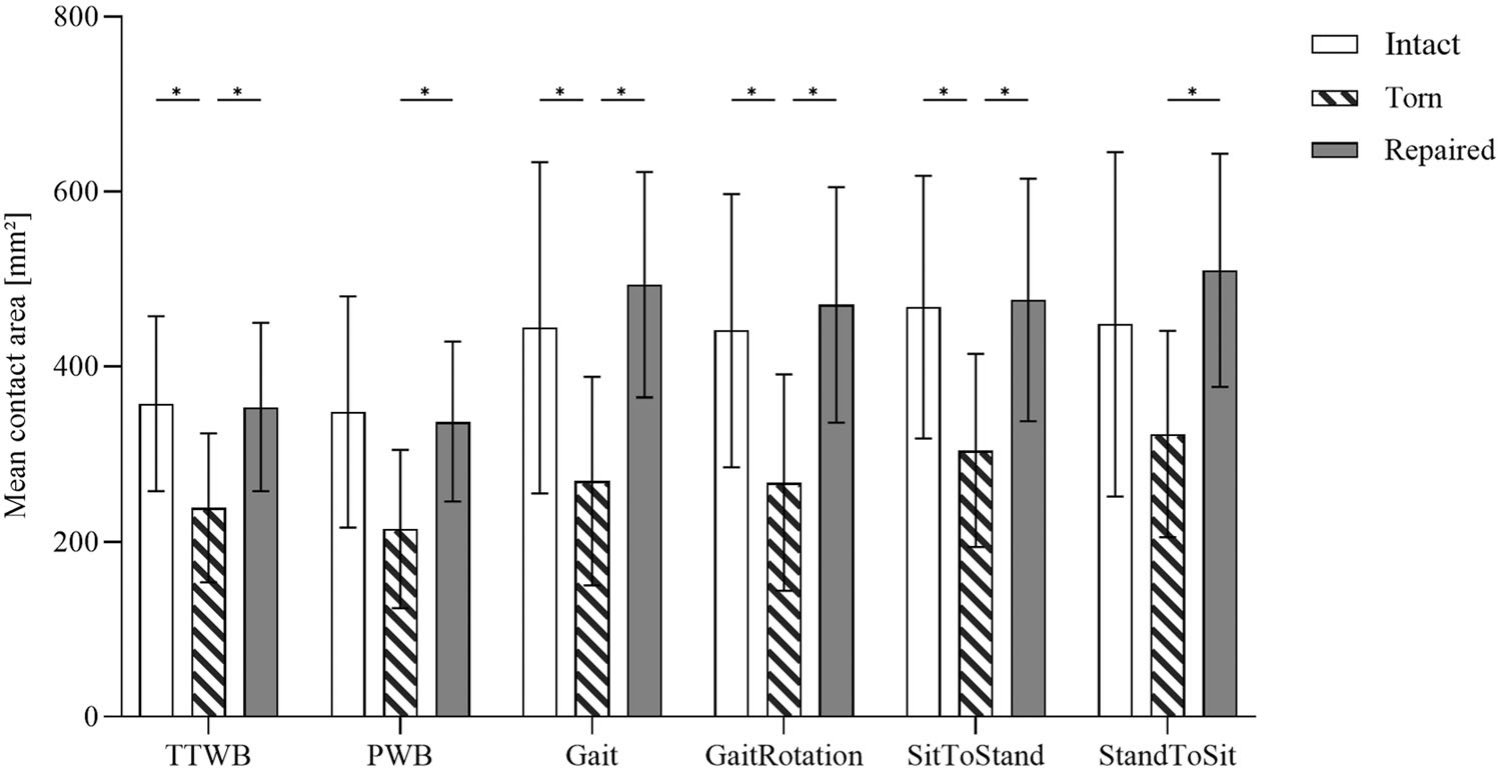
Mean contact area (mean ± SD) measurements during six movements (four‐point crutch gait under 30% BW [TTWB] and 50% BW [PWB], Gait, gait with an associated 15° ankle rotation [GaitRotation], standing up [SitToStand] and sitting down [StandToSit]), for the intact (white bar), torn (hatched bar) and repaired (solid grey bar) meniscal state. One‐way analysis of variance and Tukey's multiple comparison test. BW, body weight; PWB, partial weight‐bearing; TTWB, toe‐touch weight‐bearing. *p ≤ 0.05; *n* = 8.

In the intact state, the medial contact areas were comparable for the crutch‐assisted movements (TTWB vs. PWB) and increased under full BW simulations like GaitRotation (+26%) or sitting (+29%) (Figure 4; Statistical details: Supporting Information S2: 2B). MMPRA simulation resulted in a significant area reduction in all movements, except for PWB (−38.2%) and sitting (−28.1%). The contact area decreased by 40% during both Gait (*p* = 0.02) and GaitRotation (*p* = 0.03), and 35% (*p* = 0.02) during standing simulations. MMPRA repair was sufficient to increase the contact area comparable to the intact state in all movement simulations.

### Knee joint kinematics

**Figure 5:**
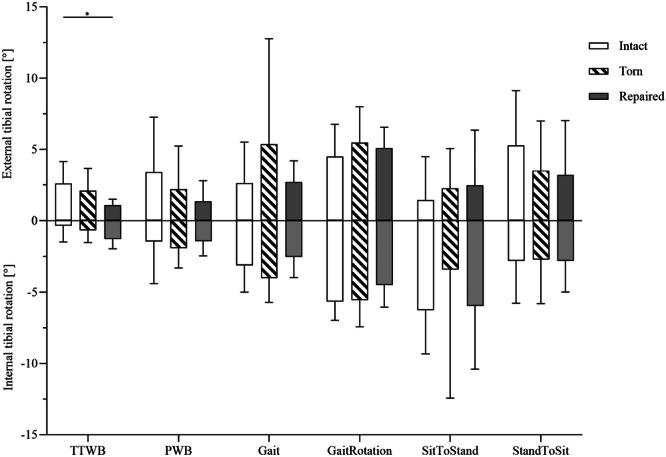
Range of motion in internal (mean − SD) and external (mean + SD) tibial rotation measurements during six movements (four‐point crutch gait under 30% BW [TTWB] and 50% BW [PWB], Gait, gait with an associated 15° ankle rotation [GaitRotation], standing up [SitToStand] and sitting down [StandToSit]), for the intact (white bar), torn (hatched bar) and repaired (solid grey bar) meniscal state. Mixed model analysis and Tukey's multiple comparison test. BW, body weight; PWB, partial weight‐bearing; TTWB, toe‐touch weight‐bearing. *p ≤ 0.05; *n* = 8.

In the intact state, the minimum external rotation was measured during standing (1.1°), while the maximum rotation was measured during simulation of the sitting movements (4.9°; +445%). Internal rotation was in the intact state lowest for the gait simulation and increased 34‐fold compared to the standing simulation (Figure 5). After MMPRA application, a slight reduction in the total ROM (combined external and internal rotation) was noted in the crutch‐assisted (TTWB and PWB) and standing movement simulations. Suture MMPRA repair resulted in a slightly lower ROM during TTWB and PWB compared to the intact state, while the ROM of the daily movement simulations (Gait, GaitRotation, sitting and standing) were restored comparable to the intact state.

### Medial meniscus posterior horn displacement

**Figure 6:**
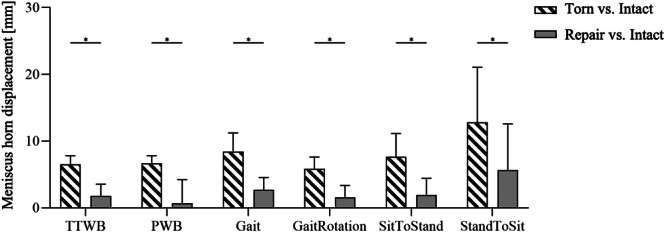
Medial displacement (mean ± SD) of the dissected posterior horn after MMPRA simulation and subsequent repair during six movements (four‐point crutch gait under 30% BW [TTWB] and 50% BW [PWB], Gait, gait with an associated 15° ankle rotation [GaitRotation], standing up [SitToStand] and sitting down [StandToSit]). Mixed model analysis and Bonferroni's multiple comparisons test. BW, body weight; MMPRA, medial meniscus posterior root avulsion; PWB, partial weight‐bearing; TTWB, toe‐touch weight‐bearing. *p ≤ 0.05; *n* = 5.

After MMPRA simulation, the horn displacement was minimal and comparable for the crutch‐assisted movements (TTWB and PWB) and gait with associated rotation movement simulations, while sitting resulted in a significant (p < 0.04) 97% and 91% increase of the medial displacement compared to TTWB and PWB, respectively (Figure 6; further details: Supporting Information S2: 2C). MMPRA repair resulted in a significantly decreased horn displacement for all movement simulations (*p* < 0.001), while the highest post‐repair displacement was measured during standing (2 mm) and sitting (5.7 mm) movements.

### Repair suture forces

**Figure 7:**
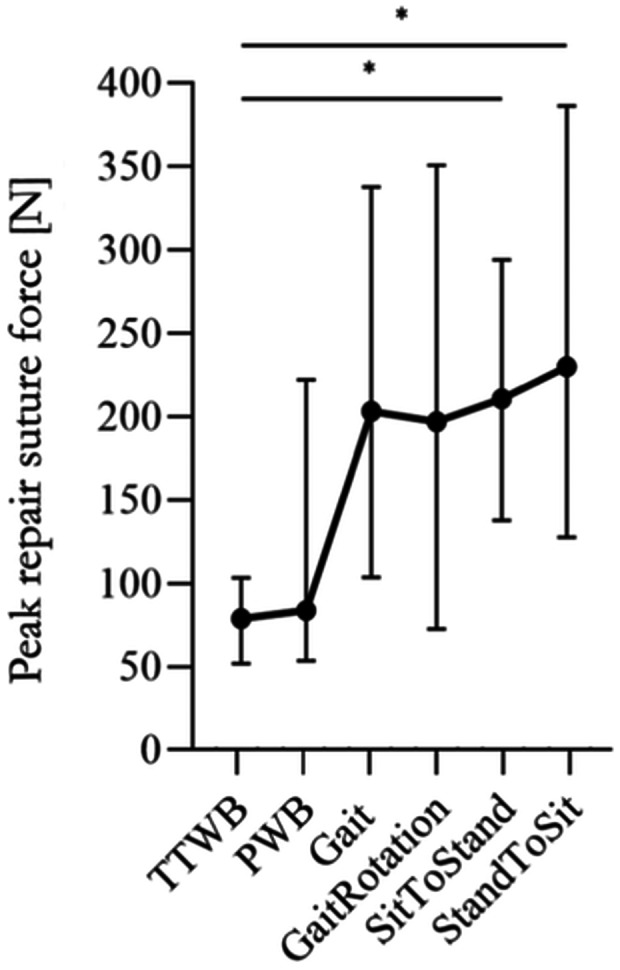
Peak repair suture force (min, median, max) in N during six movements (four‐point crutch gait under 30% BW [TTWB] and 50% BW [PWB], Gait, gait with an associated 15° ankle rotation [GaitRotation], standing up [SitToStand] and sitting down [StandToSi]). Friedmann test. BW, body weight; PWB, partial weight‐bearing; TTWB, toe‐touch weight‐bearing. *p ≤ 0.05, *n* = 7.

The median peak suture force values ranged from 77 N (TTWB) up to 232 N (sitting) (Figure 7). Accordingly, the following increase rates were measured, relative to the TTWB force: PWB—18%; Gait—152%; GaitRotation—144%; SitToStand—161% (*p* = 0.03); and StandToSit—201% (*p* = 0.001).

## DISCUSSION

The most important finding of this study is that both types of crutch‐assisted movements significantly reduced the load on the medial meniscus posterior root suture in comparison to unrestricted, daily movement simulations. Furthermore, they did not increase contact pressure, even in the presence of a MMPRA. In contrast, the unrestricted movements which exhibited deeper knee flexions caused the femoral condyles to migrate towards the posterior aspect, thus increasing the contact pressure at posterior meniscus horn area [42]. In the case of an MMPRA, the meniscus loses its ability to convert axial loads into circumferential hoop stress, while the femur condyle exerts localized pressure on the tibial plateau [45]. Our results indicated that compared to the intact state, MMPRA significantly reduced the tibiofemoral contact area in the medial compartment, except in PWB, and increased the tibiofemoral contact pressure accordingly, except in TTWB and PWB. MMPRA repair was able to increase the tibiofemoral contact area similar to that of the native state [1, 7, 45, 46, 53, 54] and restore the tibiofemoral contact pressure [53, 68], thus corroborating our first hypothesis. Furthermore, the tibiofemoral contact mechanics investigation indicated an increase in contact pressure from crutch‐assisted movements with limited BW loading (TTWB and PWB) compared to daily movements (Gait, GaitRotation, StandToSit and SitToStand) under full BW. In conclusion, by limiting tibiofemoral contact pressure as seen during TTWB and PWB, the risk of cartilage abrasion and consequently the onset of early knee joint OA [9, 58, 72], as well as the risk of post‐operative MMPRA suture failure [8, 38] might be reduced, thus corroborating our second hypothesis. Moreover, this implicates being biomechanically beneficial for patients in the immediate post‐traumatic MMPRA repair phase as well as during the weight‐bearing tolerance phase, 6 weeks postoperatively [49]. Moreover, the crutch‐assisted movements minimize the tibiofemoral contact pressure by maintaining the lower limb muscle activity, thus preventing the limb from excessive muscle atrophy.

Knee joint kinematics did not change significantly with different meniscus states. Allaire et al. observed in their study an increased external tibial rotation in the MMPRA state under axial compression of 1000 N in static knee joint flexion states [1]. In a study investigating the effects of a lateral meniscus posterior root tear on the rotational stability of the knee joint, knee cadavers were subjected to pure rotational moments of 5 N m. The authors concluded that a lateral meniscus posterior root tear can result in significant internal rotational instability within knee flexion ranges from 30° to 90° [57]. Most publications involving in vitro investigations indicate in their limitations the lack of active muscle force simulation in their studies. In contrast, our knee joint simulator utilizes seven electromechanical actuators that allow for physiologic muscle force simulation of the nine most relevant knee‐spanning muscles [57]. The interplay of these active muscle simulations does not only result in more realistic tibiofemoral contact mechanics [62] but also in enhanced overall knee joint stability when compared to in vitro investigations where only dead‐weights or quasi‐static loads were used for the application of moments and forces [68]. This is further underlined by different authors who have shown that cadaveric knee joints are significantly stabilized by active muscle force simulation [43, 70]. Therefore, we assume that the active muscle force simulation stabilized the knee joints in our study to such an extent that the kinematic alterations caused by MMPRA were only minor.

The forces on the sutures at the posterior horn are in agreement with the tibiofemoral contact pressure evolution in this study. TTWB and PWB indicated the lowest forces, followed by normal gait and gait with tibial rotation, and maximum suture forces arose during movements characterized by higher flexion levels such as standing up and sitting down. The peak forces found in the present study agree with the findings of Stärke et al. [64], who found increasing tensile forces of up to 60 N with increasing knee flexion angles. While Stärke et al. applied quasistatic loads of 100 N and 500 N, combined with external and internal rotation moments of 5 N m to their cadavers, the present study simulated tibiofemoral loading via active muscle simulation. The more dynamic knee joint loading resulted in mean peak forces of up to 232 N, as seen during the simulation of sitting down. However, during StandToSit simulation, maximum suture forces at the posterior horn reached values of up to 360 N, thus being in a critical failure region as in the load‐to‐failure in vitro test conducted by LaPrade et al. [34] and Anz et al. [2], where ultimate failure loads of up to 320 N [34] and 368 N [2], respectively, after cyclic loading were measured. These results with the associated high suture forces in kneeling movements align with findings from studies where posterior root tears frequently occurred traumatically in societies with a kneeling‐associated floor‐based lifestyle [36] and while standing up from a chair or squatting, in combination with popping sounds [3, 23]. Therefore, our results imply that unrestricted activities of daily living can lead to a highly increased risk for MMPRA suture loosening or even cut‐out suture ruptures. Contrary to Stärke et al., the force on the posterior horn was reduced in our study when a 15° external tibial rotation was applied compared to normal straight gait. However, our findings are in accordance with that of Melugin et al. [47]. They also observed a continuous decrease in force on the posterior medial meniscus root during internal knee joint rotation (in our case: external tibial rotation) at low flexion angles from 0° to 30° knee flexion. An increase in force compared to the non‐rotation state was only observed at higher flexions from 60° to 90°. In our GaitRotation simulation, the tibial rotation sequence was applied during stance phase, where no flexion angles higher than 30° are applied. As the femoral condyles glide along the tibial plateau when the knee is flexed deeply, the meniscus loses a certain amount of the initial tension at low flexion angles, even under the tibial external rotation. This tension increases again at higher flexion angles, and external tibial rotation influences the root force. In light of this finding, Melugin et al., as well as our data, do not anticipate any major concern in rehabilitation protocols in the early postoperative period for limited flexion gait walking with a moderate 15° external tibial rotation, because such a movement combination is considered to be a higher risk for MMPRA [14]. However, immediate partial WB under cyclic loading leads to meniscus micromotion [6, 12, 34], which has been identified as a key reason for fixation strength loosening [27]. Moreover, a study by Lee et al. [37] showed good clinical outcomes with only 5% (1/21) re‐tearing root repairs when avoiding partial WB for 6 weeks postoperatively, which is in line with current rehabilitation recommendations [49]. Recent clinical studies have shown that weight loss, younger age, lower BMI, and a lower hip‐knee‐angle are positively correlated with the outcomes after an MMPRA repair.

The findings of this biomechanical in vitro study to protect MMPRA‐injured and MMPRA‐repaired knees during the early post‐operative phase from loads as seen during normal gait or standing up and sitting down is further supported by the results of the RSA‐related displacement analysis on the medial posterior horn: All applied movements caused a medial displacement between 5.9 and 12.8 mm of the posterior horn after MMPRA simulation. In a prior study by Sukopp et al., radial meniscus tears indicated a gapping of up to 6 mm, which could be critical because of a potentially compromised healing [67]. Indeed, MMPRA tears are different from meniscus tears in the meniscus body because they are instead ligamentous ruptures. However, also for tendon repairs, its secure fixation to the bone is critical for clinical success [10]. Except for the StandToSit movement, MMPRA repair was able to reduce the residual displacement to <3 mm, which is considered to be a threshold for compromised meniscal healing function [63]. Therefore, distinct disparities from typical daily movements were evident, suggesting the necessity for immediate postoperative abstention from deep flexion daily movements such as standing up and sitting down under full BW loading. These biomechanical findings suggest the implementation of partial weight bearing in the early post‐operative period with a gradual transition to full weight bearing [26], which appears to be essential to promote adequate soft tissue healing in the case of MMPRA [5, 6, 18], because the hoop stress caused by weight bearing can improve meniscal healing in meniscal repair [71]. Moreover, recent clinical studies emphasized that weight loss, a lower BMI, younger patient age, and a lower hip‐knee‐angle are positively correlated with medial meniscus extrusion after an MMPRA repair, thus leading to better clinical outcomes [22, 50].

It is important to consider the limitations of the present study. First, ex vivo testing is not able to include biological processes such as initial or advanced healing, which might lead to alterations in the initial suture stability. Therefore, the tests represent a direct postoperative state after MMPRA repair, which appears to be the most critical time point to set the direction for the clinical outcome [30]. Second, previous studies have shown that the removal of skin, subcutaneous fat, and muscles does not affect the outcome measures [69]. However, the performed posteromedial capsulotomy for pressure‐sensitive film insertion may alter the knee joint stability, leading to a higher instability. In contrast to other studies explicitly investigating the rotational stability of the knee joint after MMPRA [1, 44], we did not observe kinematic differences after MMPRA simulation, suggesting appropriate rotational stability due to the simulated muscle activity. In addition, pathophysiologic changes in muscle strength following injury may lead to altered pain‐adapted muscle activation profiles, which may also affect knee kinematics. These effects could not be captured or evaluated in the established motion analysis and inverse dynamic study due to the characteristics of the healthy volunteers and study population, as well as the underlying inverse dynamic calculation profiles.

## CONCLUSION

This is the first biomechanical in vitro study on human knee joints simulating loading under daily activities and crutch‐assisted PWB movements by applying physiologic muscle force profiles in the direct postoperative state of MMPRA. Compared to daily activities under full BW, the crutch‐assisted movements with lower weight bearing and limited ROM lead to a significantly reduced repair suture force and less displacement of the posterior root. In conclusion, based on our data, the application of half BW with PWB immediately postoperatively appears unproblematic, and unintentional exceeding of advised weight‐bearing (TTWB) is unlikely to have negative short‐term consequences on suture stability and healing outcomes.

## AUTHOR CONTRIBUTIONS

Andreas Martin Seitz, Matthias Sukopp and Anita Ignatius conceived the study. Matthias Sukopp, Nina Schwab and Julian Frey performed the specimen preparation and biomechanical testing. Thomas Kappe performed the surgical treatment of the knee specimens. Jonas Schwer and Matthias Sukopp performed data analysis. Matthias Sukopp and Nina Schwab carried out the evaluation and statistics. Jonas Walter Metzger and Matthias Sukopp further developed the test setup. Mario Perl and Daniel Vogele supported with RSA evaluation. Firooz Salami conducted and helped with the MAID study. Matthias Sukopp and Nina Schwab drafted the manuscript. Andreas Martin Seitz revised the manuscript. All authors read and approved the final manuscript.

## CONFLICT OF INTEREST STATEMENT

The authors declare no conflict of interest.

## ETHICS STATEMENT

IRB 326/19, Ulm University.

## Supporting information

Supporting information.

Supporting information.

Supporting information.

Supporting information.

Supporting information.

Supporting information.

Supporting information.

Supporting information.

Supporting information.

Supplementary Information

## Data Availability

The raw data sets generated during and/or analysed during the current study are available from the corresponding author upon reasonable request.
