# Supplementary material for: Partial weight‐bearing and range of motion limitation significantly reduce the loads at medial meniscus posterior root repair sutures in a cadaveric biomechanical model
Source: Knee Surg Sports Traumatol Arthrosc. 2024 Sep 17;33(5):1645–57. doi: 10.1002/ksa.12465 (PMC12022837; doi:10.1002/ksa.12465)
Supplement: Supplementary file 9 — Supporting information. [file KSA-33-1645-s002.docx]

**Supplementary 1 – Methods Details**

1. **Specimen preparation – Transtibial pull-out repair of MMPRA (LaPrade type 2)**

MMPRA repair was performed using a transtibial bone tunnel that was drilled using a guide wire placed with a standard arthroscopic root repair aiming device into the native footprint of the MMPR. Exposure was facilitated via a posterior capsulotomy. A cannulated 4 mm reamer was then used to create the bone tunnel, the extraarticular aperture being located at the anteromedial tibial head as permitted by the fixation techniques of the tibia, at a variable angle of approximately 60 degrees. Two sutures were placed into the avulsed posterior horn of the meniscus in a double double-locking loop (D-DLL) fashion. The sutures were then pulled out through the centre of the bone tunnel and attached to the force transducer. Special care was taken, that the sutures did not touch the drill rim throughout any movement simulations.

1. **RSA marker displacement calculation)**

The methodology employed in this study involved the application of the orthogonal projection technique (Equation 1, Figure 1). The displacement of a marker bead $P$ (location $\vec{p}$), inserted in the posterior horn, was measured relative to a medially fixed coordinate system spanning a sagittal plane $E$ along the medial compartment. To calculate the change in distance, an imaginary vertical auxiliary line $h$ is projected through the marker point $\vec{p}$, orthogonal to the plane $E$ ($\vec{n}$: normal vector) (Equation 2). This procedure yielded an intersection point, denoted as $\vec{s}$, on the plane (Equation 3). The distance $d$ was calculated from the magnitude of the vector ($\vec{SP}$) (Equation 4, Equation 5). Consequently, a single tantalum marker bead was positioned within the ligamentous transition zone at the posterior horn, in close proximity to the tear site, and biplanar RSA images were acquired. To compare outcomes pre- and post-repair, an image in the intact state served as the baseline. In addition, RSA images were captured before the first and after the last cycle of each movement to assess the impact of this movement on medial translation.

| $E:\left( \vec{x}-\vec{a} \right)\cdot\vec{n}=0$ | Equation 1 |
| --- | --- |
| $h:\vec{x}=\vec{p}+\lambda\vec{n}$ | Equation 2 |
| $\vec{s}=\vec{p}+\lambda\vec{n}$ | Equation 3 |
| $\vec{SP}=\vec{p}-\vec{s}$ | Equation 4 |
| $d=\left\vert\vec{SP} \right\vert$ | Equation 5 |
| 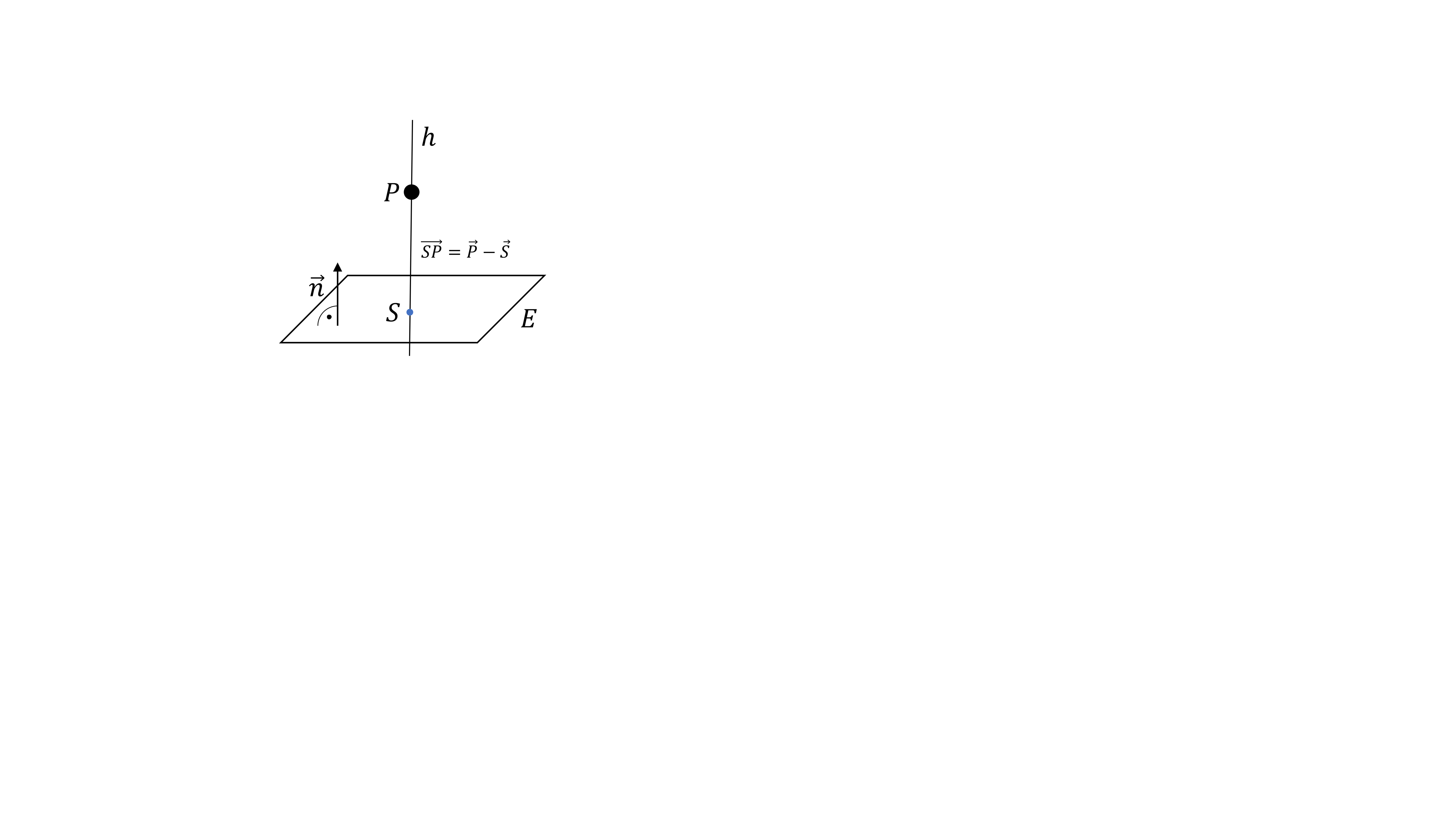 | |
| Figure 1: Illustration of the mathematical calculation of the distance between the point P and the plane E. | |
