# Supplementary material for: Partial weight‐bearing and range of motion limitation significantly reduce the loads at medial meniscus posterior root repair sutures in a cadaveric biomechanical model
Source: Knee Surg Sports Traumatol Arthrosc. 2024 Sep 17;33(5):1645–57. doi: 10.1002/ksa.12465 (PMC12022837; doi:10.1002/ksa.12465)
Supplement: Supplementary file 10 — Supplementary Information [file KSA-33-1645-s004.docx]

**Supplementary 2 – Results Details**

1. **Medial tibiofemoral mean contact pressure**

Table 1: Summary statistics and group differences (intact, torn, repaired) for the mean contact pressure measurements during six movements (four-point crutch gait under 30% BW (TTWB) and 50% BW (PWB), Gait, gait with an associated 15° ankle rotation (GaitRotation), standing up (SitToStand) and sitting down (StandToSit)); CI: confidence interval, Mean Diff: mean group difference. One-way analysis of variance and Tukey’s multiple comparison test, * p≤0.05

|  | **Tukey's multiple comparisons test** | **Mean Diff.** | **95% CI** | **Adjusted P Value** |
| --- | --- | --- | --- | --- |
|  |  |  |  |  |
| **TTWB** | Intact vs. Torn | 0.00 | -0.38 to 0.39 | >0.99 |
|  | Intact vs. Repaired | 0.16 | -0.16 to 0.49 | 0.36 |
|  | Torn vs. Repaired | 0.16 | -0.061 to 0.38 | 0.15 |
|  |  |  |  |  |
| **PWB** | Intact vs. Torn | -0.01 | -0.37 to 0.35 | >0.99 |
|  | Intact vs. Repaired | 0.17 | -0.18 to 0.51 | 0.39 |
|  | Torn vs. Repaired | 0.17 | 0.024 to 0.32 | 0.03* |
|  |  |  |  |  |
| **Gait** | Intact vs. Torn | -0.25 | -0.66 to 0.15 | 0.22 |
|  | Intact vs. Repaired | 0.16 | -0.078 to 0.40 | 0.19 |
|  | Torn vs. Repaired | 0.41 | 0.15 to 0.68 | 0.01* |
|  |  |  |  |  |
| **GaitRotation** | Intact vs. Torn | -0.17 | -0.66 to 0.32 | 0.58 |
|  | Intact vs. Repaired | 0.22 | -0.13 to 0.57 | 0.22 |
|  | Torn vs. Repaired | 0.39 | 0.11 to 0.68 | 0.01* |
|  |  |  |  |  |
| **SitToStand** | Intact vs. Torn | -0.32 | -0.81 to 0.17 | 0.20 |
|  | Intact vs. Repaired | 0.11 | -0.22 to 0.44 | 0.63 |
|  | Torn vs. Repaired | 0.43 | 0.14 to 0.72 | 0.01* |
|  |  |  |  |  |
| **StandToSit** | Intact vs. Torn | -0.28 | -0.61 to 0.051 | 0.09 |
|  | Intact vs. Repaired | 0.15 | -0.0067 to 0.31 | 0.06 |
|  | Torn vs. Repaired | 0.43 | 0.14 to 0.72 | 0.01* |

1. **Medial tibiofemoral mean contact pressure**

Table 2: Summary statistics and group differences (intact, torn, repaired) for the mean contact area measurements during six movements (four-point crutch gait under 30% BW (TTWB) and 50% BW (PWB), Gait, gait with an associated 15° ankle rotation (GaitRotation), standing up (SitToStand) and sitting down (StandToSit)); CI: confidence interval, Mean Diff: mean group difference. One-way analysis of variance and Tukey’s multiple comparison test, * p≤0.05

|  | **Tukey's multiple comparisons test** | **Mean Diff.** | **95% CI** | **Adjusted p-value** |
| --- | --- | --- | --- | --- |
|  |  |  |  |  |
| **TTWB** | Intact vs. Torn | 119 | 6.8 to 231 | 0.04* |
|  | Intact vs. Repaired | 3.9 | -97 to 105 | >0.99 |
|  | Torn vs. Repaired | -115 | -214 to -16 | 0.03* |
|  |  |  |  |  |
| **PWB** | Intact vs. Torn | 134 | -12 to 279 | 0.07 |
|  | Intact vs. Repaired | 11 | -136 to 158 | 0.97 |
|  | Torn vs. Repaired | -123 | -209 to -36 | 0.01* |
|  |  |  |  |  |
| **Gait** | Intact vs. Torn | 175 | 27 to 323 | 0.02* |
|  | Intact vs. Repaired | -49 | -242 to 144 | 0.75 |
|  | Torn vs. Repaired | -224 | -373 to -75 | <0.01* |
|  |  |  |  |  |
| **GaitRotation** | Intact vs. Torn | 174 | 17 to 331 | 0.03* |
|  | Intact vs. Repaired | -29 | -209 to 150 | 0.88 |
|  | Torn vs. Repaired | -203 | -346 to -61 | <0.01* |
|  |  |  |  |  |
| **SitToStand** | Intact vs. Torn | 164 | 25 to 302 | 0.02* |
|  | Intact vs. Repaired | -8.1 | -173 to 157 | 0.99 |
|  | Torn vs. Repaired | -172 | -293 to -50 | 0.01* |
|  |  |  |  |  |
| **StandToSit** | Intact vs. Torn | 125 | -78 to 329 | 0.23* |
|  | Intact vs. Repaired | -62 | -265 to 142 | 0.66 |
|  | Torn vs. Repaired | -187 | -306 to -68 | <0.01* |

1. **Medial meniscus posterior horn displacement**

The MMPRA repair significantly reduced the medial horn displacement to almost native levels in all movements. TTWB simulation resulted in a residual displacement of 1.8 mm (p <0,001; 95% CI: 2.2 - 7.4 mm) and PWB of 0.7 mm (p <0,001; 95% CI: 3.4 - 8.5 mm), representing an overall reduction of 72.3% and 89.6%, respectively. The residual displacement after repair increased for normal gait (Gait) is 2.7 mm (p <0,001; 95% CI: 3.4 - 8.1 mm) and for GaitRotation 1.6 mm (p <0,001; 95% CI: 1.7 - 6.9 mm). Consistent with previous findings of the present study, the highest post-repair translations were measured during standing (SitToStand: 2 mm, p <0,001; 95% CI: 3.1 - 8.3 mm) and were still critical at 5.7 mm (p <0,001; 95% CI: 4.6 - 9.8 mm) as seen during StandToSit simulations.

1. **Repair suture forces**

The repair suture amplitudes (MaxValue – MinValue; Figure 1, Table 3) gradually accelerates significantly from the two crutch-assisted movements (PWB: 34.6 ± 19.4 N, TTWB: 41.2 ± 24.6 N) to the normal gait (Gait: 152.1 ± 56.6 N, GaitRotation: 140.6 ± 71.9 N) as well as the sitting movement (StandToSit: 158.2 ± 40.6 N) to a maximum suture force increase of 466.2% detected during the standing-up movement (SitToStand: 195.9 ± 96.3 N) compared to PWB and up to 375.5% in comparison to TTWB.


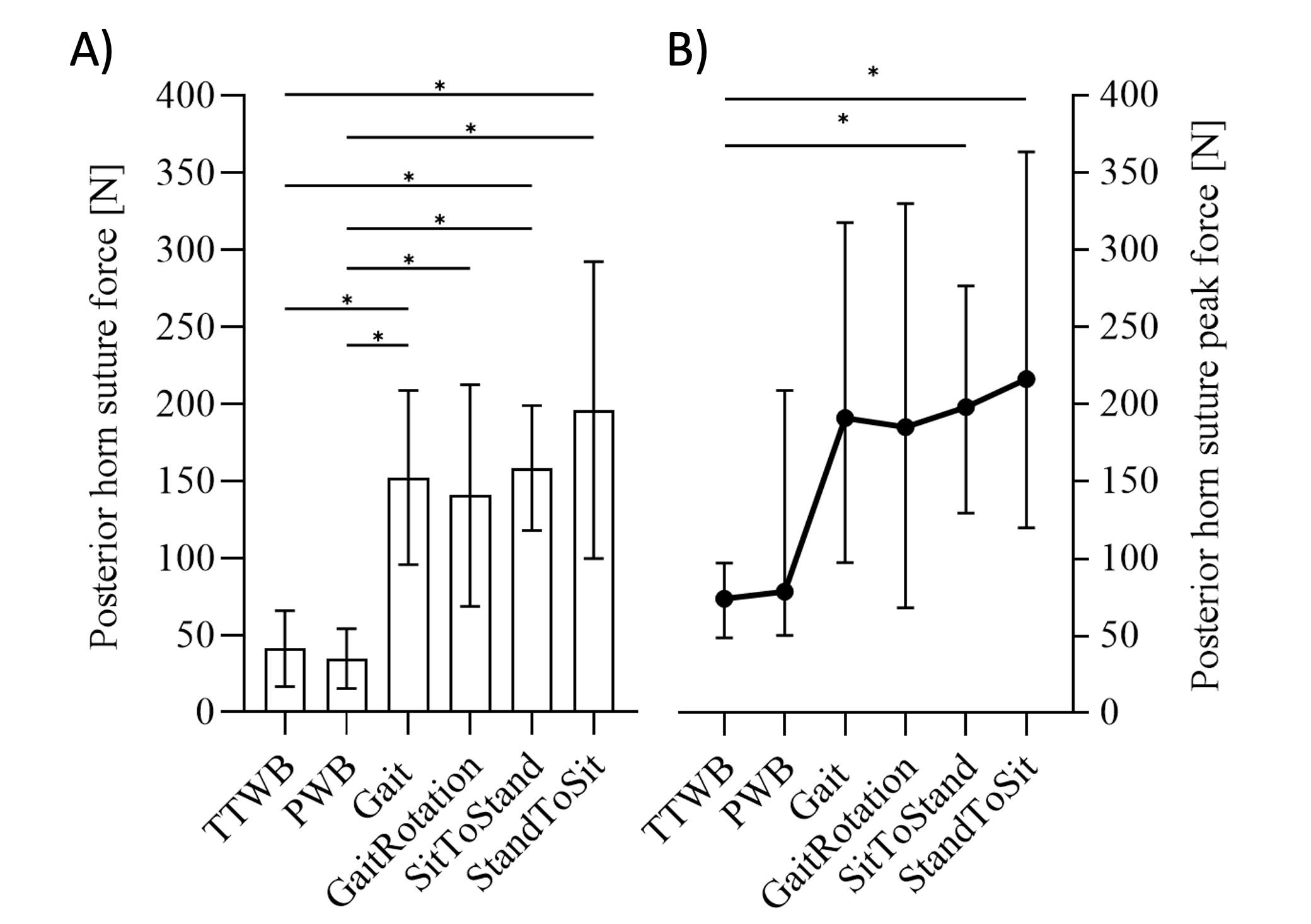


*Figure 1: Repair suture amplitudes (mean ± SD) in N at the posterior horn of the medial meniscus during six movements (four-point crutch gait under 30% BW (TTWB) and 50% BW (PWB), Gait, gait with an associated 15° ankle rotation (GaitRotation), standing up (SitToStand) and sitting down (StandToSit). One-way ANOVA and Tukey’s multiple comparison test; *p ≤ 0.05, n = 7.*

Table 3: Summary statistics and group differences for the suture repair force amplitude (MaxVal-MinVal) during six movements (four-point crutch gait under 30% BW (TTWB) and 50% BW (PWB), Gait, gait with an associated 15° ankle rotation (GaitRotation), standing up (SitToStand) and sitting down (StandToSit)); CI: confidence interval, Mean Diff: mean group difference. One-way analysis of variance and Tukey’s multiple comparison test, * p≤0.05

| **Tukey's multiple comparisons test** | **Mean Diff** | **95% CI** | **Adjusted p-value** |
| --- | --- | --- | --- |
| TTWB vs. PWB | 6.6 | -16.69 to 29.87 | 0.85 |
| TTWB vs. Gait | -110.9 | -184.2 to -37.67 | 0.01* |
| TTWB vs. GaitRotation | -99.4 | -207.1 to 8.326 | 0.07 |
| TTWB vs. SitToStand | -117.1 | -184.2 to -49.88 | <0.01* |
| TTWB vs. StandToSit | -154.7 | -274.4 to -35.04 | 0.02* |
| PWB vs. Gait | -117.5 | -191.7 to -43.39 | 0.01* |
| PWB vs. GaitRotation | -106.0 | -208.4 to -3.545 | 0.04* |
| PWB vs. SitToStand | -123.6 | -184.0 to -63.29 | <0.01* |
| PWB vs. StandToSit | -161.3 | -299.6 to -23.07 | 0.03* |
| Gait vs. GaitRotation | 11.6 | -54.44 to 77.54 | 0.98 |
| Gait vs. SitToStand | -6.1 | -103.0 to 90.73 | >0.99 |
| Gait vs. StandToSit | -43.8 | -160.4 to 72.79 | 0.68 |
| GaitRotation vs. SitToStand | -17.7 | -101.9 to 66.57 | 0.95 |
| GaitRotation vs. StandToSit | -55.4 | -188.5 to 77.76 | 0.60 |
| SitToStand vs. StandToSit | -37.7 | -174.8 to 99.43 | 0.87 |
